# Early public adherence with and support for stay-at-home COVID-19 mitigation strategies despite adverse life impact: a transnational cross-sectional survey study in the United States and Australia

**DOI:** 10.1186/s12889-021-10410-x

**Published:** 2021-03-15

**Authors:** Mark É. Czeisler, Mark E. Howard, Rebecca Robbins, Laura K. Barger, Elise R. Facer-Childs, Shantha M. W. Rajaratnam, Charles A. Czeisler

**Affiliations:** 1 Turner Institute for Brain and Mental Health, Monash University, Melbourne, VIC 3800 Australia; 2 Institute for Breathing and Sleep, Austin Health, Melbourne, VIC 3084 Australia; 3 Department of Psychiatry, Brigham and Women’s Hospital, Boston, MA 02115 USA; 4 Department of Medicine, University of Melbourne, Melbourne, VIC 3010 Australia; 5 Division of Sleep and Circadian Disorders, Departments of Medicine and Neurology, Brigham and Women’s Hospital, Boston, MA 02115 USA; 6 Division of Sleep Medicine, Harvard Medical School, Boston, MA 02115 USA

**Keywords:** COVID-19, Coronavirus, SARS-CoV-2, Pandemic, Stay-at-home orders, Mitigation strategies, Mental health, Insomnia, Public health policy, Qualtrics

## Abstract

**Background:**

Governments worldwide recommended unprecedented measures to contain the coronavirus disease 2019 (COVID-19) pandemic, caused by severe acute respiratory syndrome coronavirus 2 (SARS-CoV-2). As pressure mounted to scale back measures, understanding public priorities was critical. We assessed initial public adherence with and support for stay-at-home orders in nations and cities with different SARS-CoV-2 infection and COVID-19 death rates.

**Methods:**

Cross-sectional surveys were administered to representative samples of adults aged ≥18 years from regions with different SARS-CoV-2 prevalences from April 2–8, 2020. Regions included two nations [the United States (US—high prevalence) and Australia (AU—low prevalence)] and two US cities [New York City (NY—high prevalence) and Los Angeles (LA—low prevalence)]. Regional SARS-CoV-2 and COVID-19 prevalence (cumulative SARS-CoV-2 infections, COVID-19 deaths) as of April 8, 2020: US (363,321, 10,845), AU (5956, 45), NY (81,803, 4571), LA (7530, 198). Of 8718 eligible potential respondents, 5573 (response rate, 63.9%) completed surveys. Median age was 47 years (range, 18–89); 3039 (54.5%) were female.

**Results:**

Of 5573 total respondents, 4560 (81.8%) reported adherence with recommended quarantine or stay-at-home policies (range of samples, 75.5–88.2%). Additionally, 29.1% of respondents screened positive for anxiety or depression symptoms (range of samples, 28.6–32.0%), with higher prevalences among those of younger age, female gender, and those in quarantine or staying at home most of the time versus those who did not report these behaviours. Despite elevated prevalences of adverse mental health symptoms and significant life disruptions, 5022 respondents (90.1%) supported government-imposed stay-at-home orders (range of samples, 88.9–93.1%). Of these, 90.8% believed orders should last at least three more weeks or until public health or government officials recommended, with support spanning the political spectrum.

**Conclusions:**

Public adherence with COVID-19 mitigation policies was highly prevalent, in both highly-affected (US, NY) and minimally-affected regions (AU, LA). Despite disruption of respondents’ lives, the vast majority supported continuation of extended stay-at-home orders. Despite common support, these two countries diverged in stringent mitigation implementation, which may have contributed to subsequent outcomes. These results reveal the importance of surveillance of public support for and adherence with such policies during the COVID-19 pandemic and for future infectious disease outbreaks.

**Supplementary Information:**

The online version contains supplementary material available at 10.1186/s12889-021-10410-x.

## Background

As of 7 March 2021, there have been 116 million confirmed cases of the novel severe acute respiratory syndrome coronavirus 2 (SARS-CoV-2) worldwide, which have contributed to nearly 2.6 million deaths from coronavirus disease 2019 (COVID-19) [1]. In March 2020, during the initial phase of the COVID-19 pandemic and absent widespread testing, safe and efficacious treatments or protective vaccines, isolation and quarantine were recommended worldwide for the first time in a century. SARS-CoV-2 prevalence and associated public health policies have varied across jurisdictions and changed over time, largely without systematic assessment of public responses to the crisis or the mitigation strategies. To provide policymakers with public priorities and perspectives, we conducted a transnational cross-sectional study to assess public adherence with and support for government-imposed stay-at-home orders among individuals from regions with varying SARS-CoV-2 prevalence, including two nations [the United States (US—high SARS-CoV-2 prevalence) and Australia (AU—low SARS-CoV-2 prevalence)] and two cities [New York City (NY—high SARS-CoV-2 prevalence) and Los Angeles (LA—low SARS-CoV-2 prevalence)]. The aims of this analysis were to assess the following in the initial stages of the pandemic: public adherence with and support for stringent COVID-19 mitigation policies, including stay-at-home orders; public concerns and experiences related to the pandemic and its mitigation; and mental health, including symptoms of anxiety, depression, and insomnia. We also sought to identify characteristics associated with non-support for and non-adherence with mitigation strategies and with adverse mental health symptoms during the COVID-19 pandemic.

## Methods

### Study design and recruitment

To evaluate public adherence with and support for recommended COVID-19 mitigation strategies, we collected cross-sectional surveys of nationally representative samples of respondents using demographic quota sampling [2]. Surveys were administered to online respondent panels by Qualtrics, LLC (Provo, Utah, and Seattle, Washington, US), a commercial survey company with a network of participants consisting of hundreds of suppliers. Recruitment methodologies include digital advertisements and promotions, word of mouth, membership referrals, social networks, TV & radio advertisements, and offline approaches.

Between April 2–8, 2020 (a one-week interval), respondents were recruited from regions with markedly different infection and death rates from COVID-19 (Table 1), including nationwide samples in the US (high SARS-CoV-2 prevalence) and AU (low SARS-CoV-2 prevalence), and citywide samples in the NY (high SARS-CoV-2 prevalence) and LA (low SARS-CoV-2 prevalence) metropolitan areas. Data reported from the US sample exclude respondents from the separate NY and LA samples, unless otherwise noted.

**Table 1:** Prevalence of SARS-CoV-2 cases and COVID-19 deaths

	Overall	US	NY	LA	AU
	(*N* = 5573)	(*N* = 3010)	(*N* = 507)	(*N* = 525)	(*N* = 1531)
**SARS-CoV-2 infections and deaths due to COVID-19**					
**Cumulative confirmed SARS-CoV-2 cases**					
Study midpoint (April 5, 2020)	279,443	273,808	67,552	5940	5635
Range (April 2—April 8, 2020)	192,278–369,277	187,302–363,321	51,810–81,803	4045–7530	4976–5956
**Cumulative COVID-19 deaths**					
Study midpoint (April 5, 2020)	7054	7020	2472	132	34
Range (April 2—April 8, 2020)	3867–10,890	3846–10,845	1562–4571	78–198	21–45

Country-level cumulative cases and deaths for US and AU were retrieved from World Health Organization COVID-19 Situational Reports [3–5]. City-level cumulative cases and deaths for NY and LA were retrieved from The New York Times Coronavirus (Covid-19) Data in the United States project, based on reports from state and local health agencies [6]. Given that cases and deaths from NY and LA were also counted in the US, the Overall column reports cases and deaths from the US and AU, retrieved from the WHO COVID-19 Situation Reports

### Study approval and informed consent

The study protocol was approved by the Monash University Human Research Ethics Committee (#24036) and conducted in accordance with ethical guidelines. This activity was also reviewed by the United States Centers for Disease Control and Prevention (CDC), which affirmed that the activity was conducted consistent with applicable federal law and CDC policies for the protection of human participants from research risks: 45 Code of Federal Regulations (CFR) part 46, 21 CFR part 56; 42 United States Code (USC) Section 241(d); 5 USC Section 552a; 44 USC Section 3501 et seq. Respondents were informed of the study purposes and provided informed electronic consent prior to commencement. Investigators received anonymised responses.

### Population

Target numbers of respondent-completed surveys follow: US (3000), NY (500), LA (500), AU (1500). These sample sizes were selected to obtain samples with margins of error at 95% confidence levels of ±1.8, ±4.4, ±4.4, and ± 2.5%, respectively. To be eligible to participate, respondents were required to have provided informed electronic consent and to have reported being aged ≥18 years with current residence in the specified regions. Demographic sampling quotas were implemented for age, gender, and either race and ethnicity (US, NY, LA) or ancestry (AU), based on 2010 US and 2016 Australian census national population estimates. Potential respondents likely to qualify based on demographic characteristics listed in their Qualtrics panellist profile were targeted during recruitment; demographic questions (gender, age, race, ethnicity, and ancestry) were included in the survey to confirm eligibility. Potential respondents received invitations and could opt to participate by activating a survey link directing them to the participant information and consent page preceding the survey. Ineligible respondents who did not meet inclusion criteria (eg, aged < 18 years, not a resident of a targeted region) or exceeded pre-set quotas (ie, maximum demographic characteristic quota already met) were disempanelled.

### Survey instruments

The surveys contained 86 [US, NY, LA] or 85 [adapted for AU] items, with each item requiring a response, and was designed to take approximately 15 min to complete. Respondents were required to self-report demographic characteristics and respond to questions about COVID-19 and mitigation strategies, including adherence, priorities, sources of concern, and comparisons of current lifestyle versus lifestyle between October and December 2019 (ie, before COVID-19 and COVID-19 mitigation strategies). Additional health-related questions were asked independent of COVID-19. When possible, brief validated instruments were used, including the Short-Form Sleep Condition Indicator (SCI-02) for insomnia symptom assessment, Patient Health Questionnaire-4 (PHQ-4) for anxiety and depression symptom assessment, the Perceived Stress Scale-4 (PSS-4) for perceived stress assessment, and the Mini Z for burnout symptom assessment [7–10]. When required, validated instruments were adapted, including the Horne and Östberg Morningness-Eveningness Questionnaire (MEQ) for chronotype assessment, the μshort Munich ChronoType Questionnaire (μMCTQ) for chronotype and sleep behaviour assessment, Obstructive Sleep Apnoea 50 (OSA50) for obstructive sleep apnoea risk assessment, single-item physical activity measure, and Hurt-Insult-Threaten-Scream (HITS) screening tool for domestic violence [11–16].

### Quality screening

To verify response quality, Qualtrics conducted standardised quality screening and data cleaning procedures. Techniques included algorithmic analysis for attention patterns, click-through behaviour, duplicate responses, keystroke analysis, machine responses, and inattentiveness. Country-specific geolocation verification via IP address mapping was used to ensure respondents were from the country specified in their response. Respondents who failed an attention or speed check, along with any responses identified by the data scrubbing algorithms, were excluded from the final sample.

### Statistical analysis

Descriptive summary data are reported overall and by each sample. Multivariable Poisson regression models with robust standard errors were used to estimate adjusted prevalence ratios (aPRs) and 95% confidence intervals (CIs) for mitigation behaviours adjusted for the following explanatory variables: gender, age, political ideology, and nation (US or Australia) or city (New York or Los Angeles). For the multivariable analysis, respondents who reported a gender other than Male or Female (ie, “Other,” *n* = 4 [2 in the US sample, 2 in the Australian sample]) were excluded due to small cell sizes. The nation or city variable was used to account for differences in these sample populations, including SARS-CoV-2 and COVID-19 prevalence, mitigation policies, and other cultural or regional differences. In the cities model, combined race/ethnicity was also included as an explanatory variable. Employment status and marital status were excluded from the models to avoid collinearity with age. Separate models were run with dependent variables of having not self-reported quarantine or spending most of the time at home and having not supported stay-at-home orders as dependent variables. Additional models were run to estimate aPRs and 95% CIs for anxiety or depressive disorder symptoms and for insomnia symptoms with the same explanatory variables, plus a variable indicating whether respondents self-reported having been in quarantine or spending most of time at home. Python (version 3.7.8; Python Software Foundation) and the Python statsmodels package were used to conduct all analyses. Statistical significance was determined as *p* < 0.05.

## Results

**Fig. 1:**
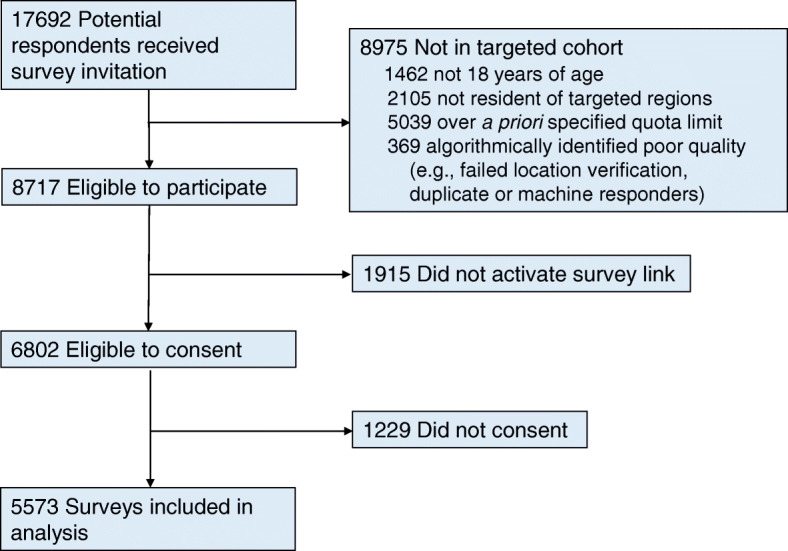
Flow of Survey Respondents. The survey was managed through an online respondent panel by Qualtrics. A priori quota limits were determined prior to study initiation to ensure nationally representative samples were collected and included the following: age, gender, and either race and ethnicity (US, NY, LA) or ancestry (AU), based on 2010 US and 2016 Australian census population estimates, respectively. Of 8718 eligible potential respondents, 5573 completed surveys, providing a 63.9% response rate

Between April 2 and April 8, 2020, of 8717 eligible invited adults, 5573 (63.9%) completed surveys (Fig. 1). The regional number of respondents, response rates, and 95% confidence level margins of error follow: US: *n* = 3010, response rate = 64.1%, margin of error = ±1.8%; NY: 507, response rate = 53.2%, margin of error = ±4.4%; LA: 525, response rate = 58.6%, margin of error = ±4.3%; AU: 1531, response rate = 70.6%, margin of error = ±2.5%. Overall, 3039 (54.5%) respondents were female; the median age of participants was 47 years (range, 18–89). Respondent demographic characteristics of categories with and without pre-specified quotas are reported in Tables 2 and 3, respectively. The state- and territory-level geographic distributions of respondents’ residency for each nationwide sample are reported in Additional file 1 online. Respondents’ personal experiences with COVID-19 and knowledge of others’ experiences with COVID-19 (Table 4) were consistent with the samples having recruited respondents with residence in regions with markedly different regional SARS-CoV-2 infection and COVID-19 death rates. NY had the highest percentage of respondents who reported knowing someone who had tested positive for SARS-CoV-2 (27.0% vs. 5.6–11.0% for the rest of the samples), or who had been hospitalised for (14.6% vs. 2.4–6.5% for the rest of the samples) or died from (9.5% vs. 0.7–2.9% for the rest of the samples) COVID-19.

**Table 2:** Self-reported respondent characteristics with pre-specified quotas

Characteristic	Overall		US^a^		NY		LA		AU	
	(*N* = 5573)		(*N* = 3010)		(*N* = 507)		(*N* = 525)		(*N* = 1531)	
Age (years)										
Mean (SD)	47.1	(17.3)	47.4	(16.9)	46.7	(18.0)	45.5	(17.0)	45.6	(17.3)
Median	47		48		45		45		44.5	
Range	18–89		18–89		18–86		18–87		18–89	
Gender—No. (%)										
Female	3039	(54.5)	1683	(55.9)	239	(47.1)	275	(52.4)	842	(55.0)
Male	2530	(45.4)	1325	(44.0)	268	(52.9)	250	(47.6)	687	(44.9)
Other	4	(0.1)	2	(0.1)	0	(0.0)	0	(0.0)	2	(0.1)
Race^b^ (All US, *N* = 4042)—No. (%)										
White	3196	(79.1)	2423	(80.5)	373	(73.6)	400	(76.2)		
Black or African American	428	(10.6)	313	(10.4)	63	(12.4)	52	(9.9)		
Asian	256	(6.3)	192	(6.4)	32	(6.3)	32	(6.1)		
American Indian or Alaskan Native	80	(2.0)	60	(2.0)	9	(1.8)	11	(2.1)		
Native Hawaiian or Pacific Islander	22	(0.5)	17	(0.6)	3	(0.6)	2	(0.4)		
Other	182	(4.5)	104	(3.5)	38	(7.5)	40	(7.6)		
Ethnicity (All US, *N* = 4042)—No. (%)										
Hispanic or Latino	424	(10.5)	265	(8.8)	69	(13.6)	90	(17.1)		
Not Hispanic or Latino	3618	(89.5)	2745	(91.2)	438	(86.4)	435	(82.9)		
Ancestry^c^ (AU, *N* = 1531)—No. (%)										
Australian									556	(36.3)
English									501	(32.7)
Other European (Irish, Scottish, German, Italian, Greek, Dutch)									346	(22.6)
Scottish									95	(6.2)
Chinese									90	(5.9)
Indian									45	(2.9)
Indigenous—Aboriginal Australians and Torres Strait Islanders									16	(1.0)
Other									455	(29.8)

Self-reported characteristics of categories with pre-specified quota limits overall and in regional samples collected in the US, NY, LA, and AU. For age, mean (standard deviation), median, and range are shown per sample. For all other characteristics, the number and percentage of respondents are reported by cohort. Race and ethnicity (US, NY, LA) or ancestry (AU) were reported in based on questions culturally adapted to match the characteristic data collected in the 2010 United States and 2016 Australian Census, respectively

^a^Respondents in the US sample do not include those who were separately recruited for the NY or LA samples, but include respondents from these cities

^b^For the US sample, respondents had the option to select more than one racial affiliation

^c^For the AU sample, respondents had the option to select up to two ancestral affiliations

The ‘Other’ category includes Filipino, Vietnamese, Lebanese, Hmong, Kurdish, Maori, and Australian South Sea Islander

**Table 3:** Self-reported respondent characteristics without pre-specified quotas

Characteristic	Overall		US^a^		NY		LA		AU	
	(*N* = 5573)		(*N* = 3010)		(*N* = 507)		(*N* = 525)		(*N* = 1531)	
Highest degree or level of education completed—No. (%)										
Less than high school	107	(1.9)	61	(2.0)	4	(0.8)	5	(1.0)	37	(2.4)
High school or equivalent	1257	(22.6)	524	(17.4)	81	(16.0)	61	(11.6)	591	(38.6)
Some college	1444	(25.9)	910	(30.2)	121	(23.9)	157	(29.9)	256	(16.7)
Bachelor’s degree (4-year) or equivalent	1806	(32.4)	927	(30.8)	159	(31.4)	212	(40.4)	508	(33.2)
Doctoral or professional degree	917	(16.5)	567	(18.8)	137	(27.0)	88	(16.8)	125	(8.2)
Prefer not to say	42	(0.8)	21	(0.7)	5	(1.0)	2	(0.4)	14	(0.9)
Marital status—No. (%)										
Married	2724	(48.9)	1567	(52.1)	231	(45.6)	226	(43.0)	700	(45.7)
Living with partner	533	(9.6)	241	(8.0)	43	(8.5)	51	(9.7)	198	(12.9)
Separated	92	(1.7)	32	(1.1)	7	(1.4)	2	(0.4)	51	(3.3)
Divorced	490	(8.8)	291	(9.7)	40	(7.9)	46	(8.8)	113	(7.4)
Widowed	178	(3.2)	109	(3.6)	12	(2.4)	21	(4.0)	36	(2.4)
Never married	1490	(26.7)	739	(24.6)	165	(32.5)	169	(32.2)	417	(27.2)
Prefer not to say	66	(1.2)	31	(1.0)	9	(1.8)	10	(1.9)	16	(1.0)
2019 household income (USD)—No. (%)										
Less than $25,000	940	(16.9)	454	(15.1)	57	(11.2)	67	(12.8)	362	(23.6)
$25,000 to $49,999	1296	(23.3)	641	(21.3)	88	(17.4)	88	(16.8)	479	(31.3)
$50,000 to $99,999	1723	(30.9)	989	(32.9)	139	(27.4)	164	(31.2)	431	(28.2)
$100,000 to $199,999	1054	(18.9)	657	(21.8)	151	(29.8)	134	(25.5)	112	(7.3)
$200,000 or more	229	(4.1)	132	(4.4)	41	(8.1)	42	(8.0)	14	(0.9)
Prefer not to say	331	(5.9)	137	(4.6)	31	(6.1)	30	(5.7)	133	(8.7)
2019 employment status—No. (%)										
Employed full-time	2245	(40.3)	1284	(42.7)	246	(48.5)	217	(41.3)	498	(32.5)
Employed part-time	760	(13.6)	338	(11.2)	63	(12.4)	61	(11.6)	298	(19.5)
Self-employed	361	(6.5)	189	(6.3)	30	(5.9)	52	(9.9)	90	(5.9)
Student	337	(6.0)	147	(4.9)	30	(5.9)	36	(6.9)	124	(8.1)
Retired	1268	(22.8)	734	(24.4)	101	(19.9)	110	(21.0)	323	(21.1)
Unemployed	714	(12.8)	384	(12.8)	45	(8.9)	55	(10.5)	230	(15.0)
Political ideology—No. (%)										
Very liberal	701	(12.6)	410	(13.6)	97	(19.1)	94	(17.9)	100	(6.5)
Slightly liberal	1121	(20.1)	586	(19.5)	107	(21.1)	129	(24.6)	299	(19.5)
Neither liberal nor conservative	1465	(26.3)	727	(24.2)	122	(24.1)	126	(24.0)	490	(32.0)
Slightly conservative	1097	(19.7)	621	(20.6)	80	(15.8)	84	(16.0)	312	(20.4)
Very conservative	701	(12.6)	484	(16.1)	60	(11.8)	58	(11.0)	99	(6.5)
Apolitical and/or prefer not to say	488	(8.8)	182	(6.0)	41	(8.1)	34	(6.5)	231	(15.1)

Self-reported characteristics of categories without pre-specified quota limits overall and in regional samples collected in the US, NY, LA, and AU. As in Table 2, the number and percentage of respondents are reported by cohort

^a^Respondents in the US sample do not include those who were separately recruited for the NY or LA samples, but include respondents from these cities

**Table 4:** Experiences with COVID-19 overall and by region

	Overall		US^a^		NY		LA		AU	
	(*N* = 5573)		(*N* = 3010)		(*N* = 507)		(*N* = 525)		(*N* = 1531)	
**Experience with COVID-19**										
Tested for SARS-CoV-2—No. (%)	119	(2.1)	56	(1.9)	18	(3.6)	11	(2.1)	34	(2.2)
Positive	10	(0.2)	5	(0.2)	4	(0.8)	0	(0.0)	1	(0.1)
Negative	88	(1.6)	36	(1.2)	13	(2.6)	11	(2.1)	28	(1.8)
Awaiting results	21	(0.4)	15	(0.5)	1	(0.2)	0	(0.0)	5	(0.3)
Not tested	5454	(97.9)	2954	(98.1)	489	(96.4)	514	(97.9)	1497	(97.8)
Hospitalized for COVID-19—No. (%)	38	(0.7)	20	(0.7)	7	(1.4)	6	(1.1)	5	(0.3)
Not hospitalized	5535	(99.3)	2990	(99.3)	500	(98.6)	519	(98.9)	1526	(99.7)
Know someone…—No. (%)										
confirmed positive with SARS-CoV-2	602	(10.8)	331	(11.0)	137	(27.0)	49	(9.3)	85	(5.6)
Colleague(s)	141	(2.5)	74	(2.5)	40	(7.9)	4	(0.8)	23	(1.5)
Family Member(s)	120	(2.2)	71	(2.4)	30	(5.9)	7	(1.3)	12	(0.8)
Friend(s)	315	(5.7)	165	(5.5)	83	(16.4)	30	(5.7)	37	(2.4)
Significant other(s)	28	(0.5)	18	(0.6)	4	(0.8)	0	(0.0)	6	(0.4)
Other	75	(1.3)	42	(1.4)	11	(2.2)	9	(1.7)	13	(0.8)
No	4971	(89.2)	2679	(89.0)	370	(73.0)	476	(90.7)	1446	(94.4)
hospitalized due to COVID-19	336	(6.0)	192	(6.4)	74	(14.6)	34	(6.5)	36	(2.4)
Colleague(s)	68	(1.2)	39	(1.3)	16	(3.2)	4	(0.8)	9	(0.6)
Family Member(s)	80	(1.4)	51	(1.7)	14	(2.8)	7	(1.3)	8	(0.5)
Friend(s)	168	(3.0)	85	(2.8)	42	(8.3)	20	(3.8)	21	(1.4)
Significant other(s)	15	(0.3)	8	(0.3)	3	(0.6)	2	(0.4)	2	(0.1)
Other	42	(0.8)	28	(0.9)	5	(1.0)	6	(1.1)	3	(0.2)
No	5237	(94.0)	2818	(93.6)	433	(85.4)	491	(93.5)	1495	(97.6)
who died due to COVID-19	158	(2.8)	86	(2.9)	48	(9.5)	13	(2.5)	11	(0.7)
Colleague(s)	27	(0.5)	13	(0.4)	9	(1.8)	1	(0.2)	4	(0.3)
Family Member(s)	15	(0.3)	7	(0.2)	6	(1.2)	1	(0.2)	1	(0.1)
Friend(s)	81	(1.5)	44	(1.5)	26	(5.1)	6	(1.1)	5	(0.3)
Significant other(s)	12	(0.2)	9	(0.3)	1	(0.2)	0	(0.0)	2	(0.1)
Other	39	(0.7)	22	(0.7)	11	(2.2)	5	(1.0)	1	(0.1)
No	5415	(97.2)	2924	(97.1)	459	(90.5)	512	(97.5)	1520	(99.3)

Survey responses are reported using descriptive statistics as indicated, including number of respondents (No.), percentage of respondents (%), mean, and standard deviation (sd). For cases in which subgroups are stratified, the percentage of the total sample is reported

^a^Respondents in the US sample do not include those who were separately recruited for the NY or LA samples, but include respondents from these cities

**Fig. 2:**
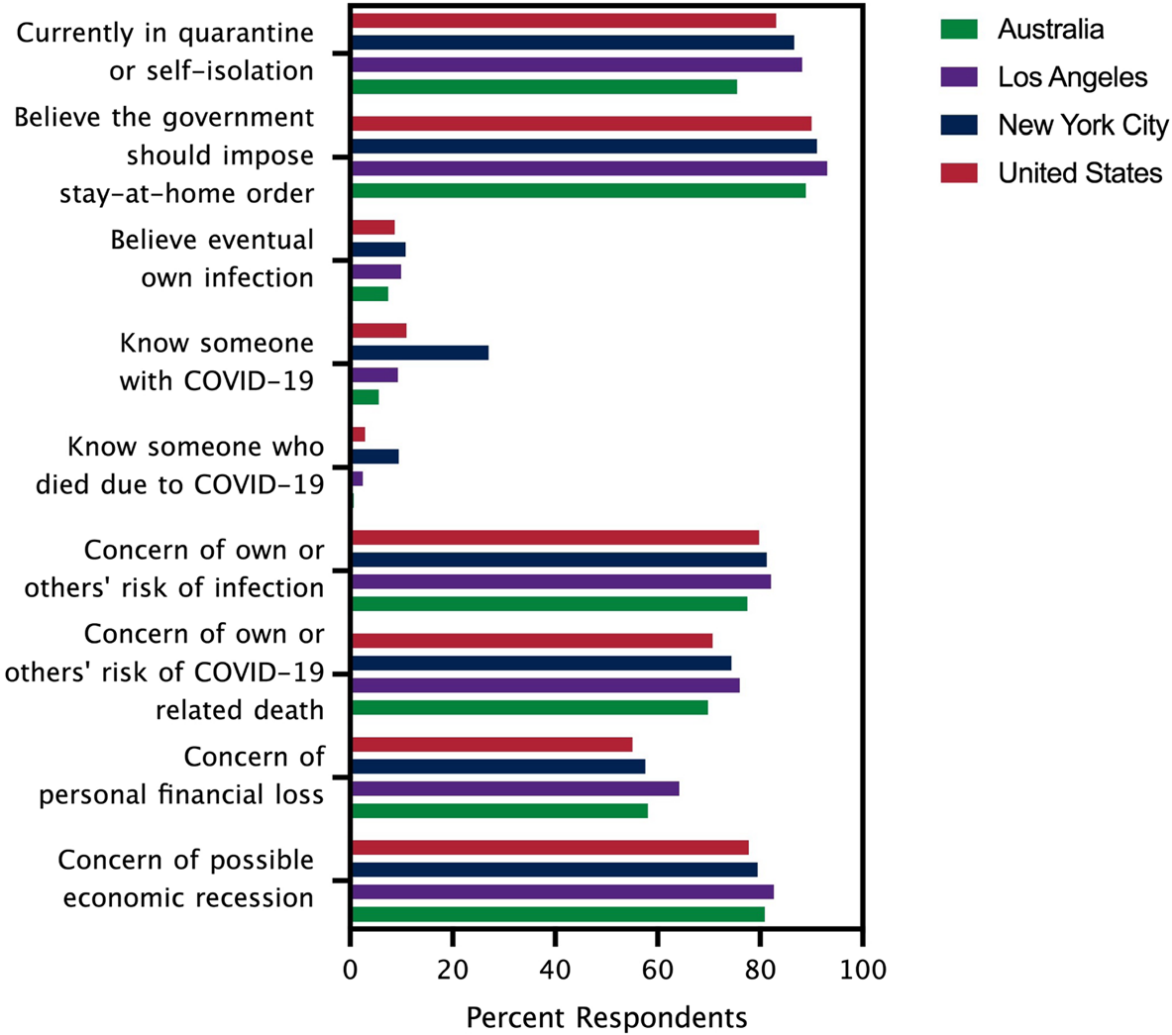
Public COVID-19 Mitigation Adherence, Concerns, Policy Support, and Experience. Percentage of respondents by region who reported: adherence with recommended mitigation strategies; support for a government-mandated stay-at-home order; perceived risk of eventual infection with SARS-CoV-2; personal experience with COVID-19 (ie, knowing someone who was infected with SARS-CoV-2 or who died from COVID-19); and moderate to extreme concerns regarding: one’s own or others’ risk of infection with SARS-CoV-2 or risk for death from COVID-19, personal financial loss, and possible economic recession

**Fig. 3:**
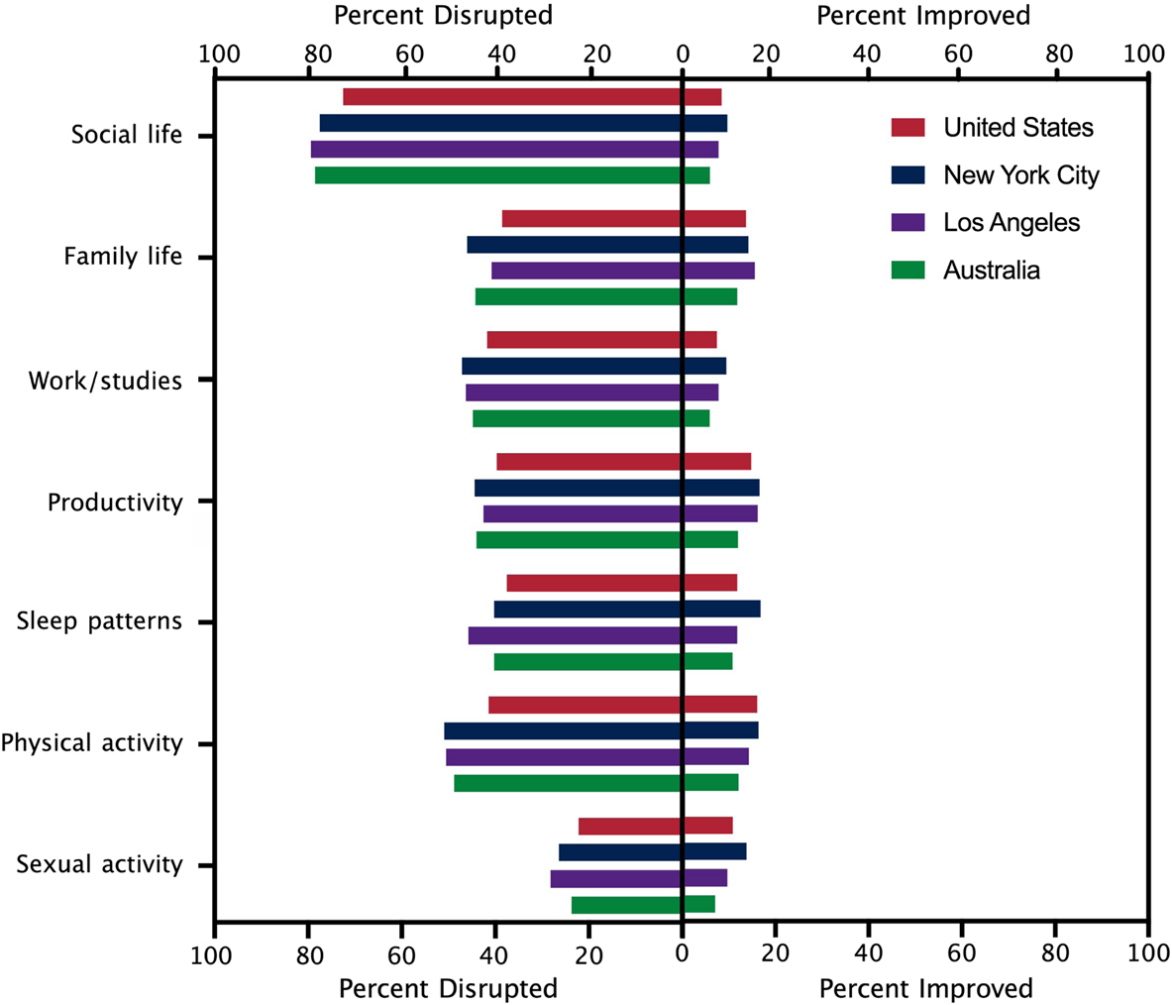
Life Disruption Due to COVID-19 and Mitigation Strategies. Impact by region of COVID-19 and mitigation strategies on social life, family life, work and/or study, productivity, sleep patterns, physical activity, and sexual activity; percentage of respondents reporting that the indicated behavioural category was moderately to extremely disrupted or improved is shown

**Fig. 4:**
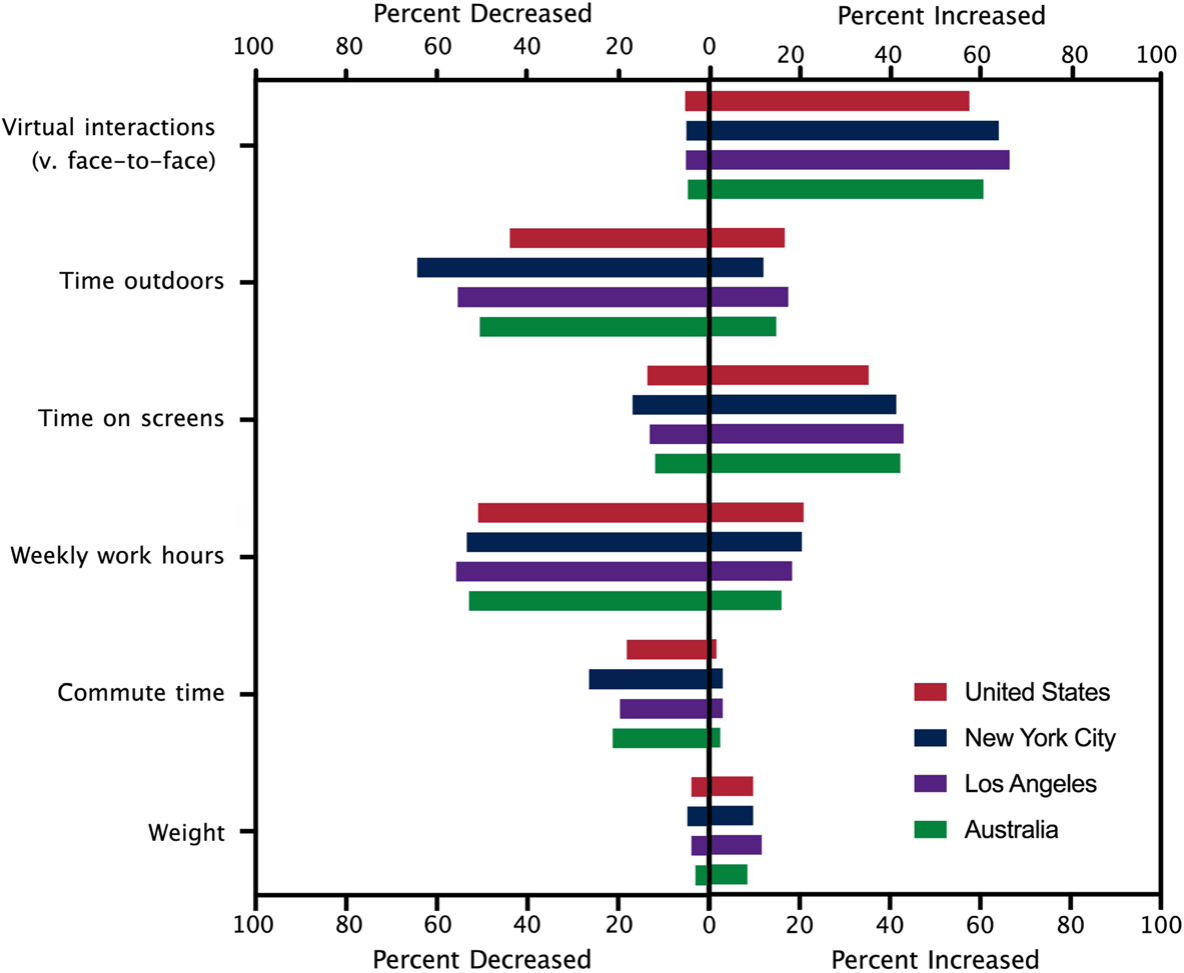
Behavioural Changes Comparing Before and During the COVID-19 Pandemic. Percentage of respondents reporting decreases or increases in six categories [virtual interactions vs. face-to-face interactions; time spent outdoors during daylight hours; time on light-emitting screens; weekly work hours (among respondents employed in the fourth quarter of 2019, *n* = 3328); commute time; and weight] at the time of the survey in April, 2020 (after the COVID-19 pandemic was declared and mitigation policies were implemented) as compared to the fourth quarter of 2019 (before the COVID-19 pandemic was declared)

Respondents’ COVID-19-mitigation adherence, public priorities, life impact, and mental health symptoms are illustrated in Figs. 2, 3, 4. Altogether, 4560 respondents (81.8%) reported having been in quarantine or spending the majority of time at home (range of samples, 75.5–88.2%) (Fig. 2, Table 5). Moreover, 5022 (90.1%) believed a government-imposed stay-at-home order was warranted (range of samples, 88.9–93.1%). Of these, 90.8% believed the order should have lasted at least 3 weeks (9.1%), a month or longer (43.8%), or until public health (31.1%) or government officials (6.8%) determined it was safe to lift the restrictions. Of 5304 respondents (95.2%) who made predictions, the average predicted date by which COVID-19 would no longer affect their daily lives was between mid-June 2020 and mid-August 2020, though there was high variability in predictions (Table 5). Strong support for government-imposed stay-at-home orders spanned the political spectrum.

**Table 5:** Adherence with, support for, and predictions about mitigation strategies

	Overall		US^a^		NY		LA		AU	
	(*N* = 5573)		(*N* = 3010)		(*N* = 507)		(*N* = 525)		(*N* = 1531)	
**Adherence with COVID-19 Mitigation Strategies**										
Neither in quarantine nor spending the majority of time at home	1013	(18.2)	508	(16.9)	68	(13.4)	62	(11.8)	375	(24.5)
In quarantine or self-isolation	4560	(81.8)	2502	(83.1)	439	(86.6)	463	(88.2)	1156	(75.5)
Quarantine^b^	1946	(34.9)	1151	(38.2)	214	(42.2)	224	(42.7)	357	(23.3)
Spending most of the time at home	2614	(46.9)	1351	(44.9)	225	(44.4)	239	(45.5)	799	(52.2)
**Predictions for When COVID-19 Will Stop Affecting Daily Life**										
Respondents offering predictions^c^—No. (%)	5304	(95.2)	2878	(95.6)	480	(94.7)	501	(95.4)	1445	(94.4)
Number of days from survey completion date—mean (sd)	92.5	99.37	76.4	84.12	79.4	77.02	78.6	77.45	134.0	125.51
Calendar Date	7/5/2020		6/16/2020		6/22/2020		6/21/2020		8/15/2020	
**Public Priorities for COVID-19 Mitigation Strategies**										
Government should impose stay-at-home order^d^—No. (%)										
No	551	(9.9)	300	(10.0)	45	(8.9)	36	(6.9)	170	(11.1)
Yes	5022	(90.1)	2710	(90.0)	462	(91.1)	489	(93.1)	1361	(88.9)
for 1 week	89	(1.6)	56	(1.9)	6	(1.2)	8	(1.5)	19	(1.2)
for 2 weeks	373	(6.7)	215	(7.1)	46	(9.1)	25	(4.8)	87	(5.7)
for 3 weeks	457	(8.2)	271	(9.0)	51	(10.1)	49	(9.3)	86	(5.6)
for at least 1 month	2201	(39.5)	1298	(43.1)	190	(37.5)	254	(48.4)	459	(30.0)
until health officials say it is safe	1562	(28.0)	737	(24.5)	133	(26.2)	127	(24.2)	565	(36.9)
until government officials say it is safe	340	(6.1)	133	(4.4)	36	(7.1)	26	(5.0)	145	(9.5)
By political affiliation										
Very liberal	701	(12.6)	410	(13.6)	97	(19.1)	94	(17.9)	100	(6.5)
No	51	(0.9)	30	(1.0)	4	(0.8)	2	(0.4)	15	(1.0)
Yes	650	(11.7)	380	(12.6)	93	(18.3)	92	(17.5)	85	(5.6)
Somewhat liberal	1121	(20.1)	586	(19.5)	107	(21.1)	129	(24.6)	299	(19.5)
No	64	(1.1)	28	(0.9)	4	(0.8)	6	(1.1)	26	(1.7)
Yes	1057	(19.0)	558	(18.5)	103	(20.3)	123	(23.4)	273	(17.8)
Neither liberal nor conservative	1465	(26.3)	727	(24.2)	122	(24.1)	126	(24.0)	490	(32.0)
No	161	(2.9)	81	(2.7)	9	(1.8)	9	(1.7)	62	(4.0)
Yes	1304	(23.4)	646	(21.5)	113	(22.3)	117	(22.3)	428	(28.0)
Somewhat conservative	1097	(19.7)	621	(20.6)	80	(15.8)	84	(16.0)	312	(20.4)
No	117	(2.1)	59	(2.0)	12	(2.4)	12	(2.3)	34	(2.2)
Yes	980	(17.6)	562	(18.7)	68	(13.4)	72	(13.7)	278	(18.2)
Very conservative	701	(12.6)	484	(16.1)	60	(11.8)	58	(11.0)	99	(6.5)
No	97	(1.7)	70	(2.3)	11	(2.2)	6	(1.1)	10	(0.7)
Yes	604	(10.8)	414	(13.8)	49	(9.7)	52	(9.9)	89	(5.8)
Apolitical/prefer not to say	488	(8.8)	182	(6.0)	41	(8.1)	34	(6.5)	231	(15.1)
No	61	(1.1)	32	(1.1)	5	(1.0)	1	(0.2)	23	(1.5)
Yes	427	(7.7)	150	(5.0)	36	(7.1)	33	(6.3)	208	(13.6)

Survey responses are reported using descriptive statistics as indicated, including number of respondents (No.), percentage of respondents (%), mean, and standard deviation (sd)

^a^Respondents in the US sample do not include those who were separately recruited for the NY or LA samples, but include respondents from these cities

^b^Quarantine was defined as “not attending public places, including work, supermarkets or pharmacies, school or childcare, places of worship, etc. Individuals in quarantine do not have visitors and only live with people who usually live in your home. They stay at home or accommodation unless medical care is required.”

^c^Predictions in the year 2030 or beyond were excluded. There were six such predictions: (US, 8/6/2064, 2/1/2071), (LA, 1/1/2030, 1/1/2032, 12/31/2050), (AU, 8/10/2066)

^d^Stay-at-home was defined as “all non-essential services, such as dine-in restaurants, bars, social venues, gyms, fitness studios, and convention centers, are shut down. Essential services, such as groceries, pharmacies, gas stations, food banks, convenience stores, and delivery restaurants, remain open. Banks, local governments that provide services, and law enforcement agencies also remain open”

In the nations model for non-adherence with mitigation policies, respondents of female versus male gender and aged 18–24 years versus ≥65 years significantly less commonly reported neither being in quarantine nor spending the majority of time at home (Table 6). Compared to those with centrist liberal ideology, liberal respondents less commonly reported non-adherence, while very conservative respondents more commonly reported this behaviour. Respondents from the US also less commonly reported non-adherence than those from Australia. In the cities model, the gender difference was also observed. No other characteristics were associated with significant differences.

**Table 6:** Characteristics associated with non-adherence with and non-support for COVID-19 mitigation measures

**Non-adherence: aPRs for neither being in quarantine nor spending most of the time at home**						
	**Nations** **US and Australia** ***n*** **= 4537**			**Cities** **New York and Los Angeles** ***n*** **= 1032**		
	**aPR**	**95% CI**	***P***	**aPR**	**95% CI**	***P***
Gender^a^ (reference: Male)						
Female	0.81	(0.72, 0.91)	0.001	0.67	(0.48, 0.93)	0.016
Age group, years (reference: ≥65)						
18–24	0.59	(0.46, 0.76)	< 0.001	0.65	(0.29, 1.44)	0.29
25–44	0.88	(0.74, 1.04)	0.13	1.29	(0.76, 2.17)	0.34
45–64	1.07	(0.91, 1.25)	0.40	1.50	(0.91, 2.46)	0.11
Political ideology (reference: Centre)						
Very Liberal	0.64	(0.50, 0.82)	< 0.001	1.05	(0.64, 1.71)	0.85
Slightly Liberal	0.64	(0.53, 0.78)	< 0.001	0.76	(0.45, 1.27)	0.29
Slightly Conservative	0.89	(0.76, 1.05)	0.18	0.95	(0.57, 1.59)	0.85
Very Conservative	0.93	(0.76, 1.13)	0.44	1.15	(0.68, 1.94)	0.59
Apolitical and/or prefer not to say	1.22	(1.00, 1.48)	0.049	1.35	(0.74, 2.46)	0.33
Region^b^ (reference: Australia and LA, respectively)						
US and NY, respectively	0.72	(0.63, 0.81)	< 0.001	1.12	(0.81, 1.54)	0.50
**Non-Support: aPRs for not supporting stay-at-home orders**						
	**Nations** **US and Australia** ***n*** **= 4537**			**Cities** **New York and Los Angeles** ***n*** **= 1032**		
	**aPR**	**95% CI**	***P***	**aPR**	**95% CI**	***P***
Gender^a^ (reference: Male)						
Female	0.67	(0.57, 0.80)	< 0.001	0.78	(0.51, 1.19)	0.25
Age group, years (reference: ≥65)						
18–24	1.83	(1.30, 2.56)	< 0.001	1.44	(0.58, 3.56)	0.43
25–44	1.71	(1.29, 2.27)	< 0.001	1.42	(0.74, 2.73)	0.29
45–64	1.73	(1.31, 2.29)	< 0.001	1.53	(0.81, 2.86)	0.19
Political ideology (reference: Centre)						
Very Liberal	0.77	(0.56, 1.07)	0.12	0.44	(0.18, 1.07)	0.070
Slightly Liberal	0.53	(0.39, 0.72)	< 0.001	0.66	(0.31, 1.41)	0.29
Slightly Conservative	0.90	(0.70, 1.15)	0.40	2.14	(1.20, 3.83)	0.010
Very Conservative	1.24	(0.96, 1.60)	0.11	2.04	(1.09, 3.82)	0.027
Apolitical and/or prefer not to say	1.13	(0.84, 1.53)	0.41	0.96	(0.40, 2.29)	0.92
Region^b^ (reference: Australia and LA, respectively)						
US and NY, respectively	0.90	(0.75, 1.09)	0.28	1.27	(0.84, 1.93)	0.25

^a^For the multivariable analysis, respondents who reported a gender other than Male or Female (i.e., “Other,” *n* = 4 [2 in the US sample, 2 in the Australian sample]) were excluded due to small cell sizes

^b^Regional reference groups were chosen to represent estimated prevalence ratios for dependent variables in high versus low SARS-CoV-2 prevalence regions

In the nations model, the gender effect was similar for non-support for stay-at-home orders, with female significantly less commonly having reported not supporting such measures (aPR = 0.67, 95% CI = 0.57–0.80, *p* < 0.001) (Table 6). However, the age effect was reversed, with all younger age groups more commonly reporting non-support for stay-at-home orders than those aged ≥65 years (eg, 18–24 years, 1.83, 1.30–2.56, *p* < 0.001). In the city samples, many of the aPRs are of similar magnitude and direction to the nation samples, though statistical significance was not reached. However, in contrast to the nationwide samples, in the cities model, both slightly and very conservative respondents had more than 2-fold increased prevalence of non-support than those with centred political ideology. Statistically significant differences in non-support for stay-at-home orders were not observed regionally.

Broad support for stringent mitigation policies was reported despite elevated prevalences of adverse mental health symptoms compared with pre-pandemic estimates using similar screening instruments. Overall, 1303 respondents (23.4%, range of samples, 22.1–25.4%) screened positive for symptoms of an anxiety disorder and 1172 (21.0%, range of samples, 20.0–22.7%) screened positive for symptoms of a depressive disorder, with 1622 participants (29.1%, range of samples, 28.6–32.0%) having screened positive for symptoms of at least one of these adverse mental health conditions (Table 7). Moreover, 1029 respondents (18.5%, range of samples, 15.2–20.0%) screened positive for insomnia symptoms.

**Table 7:** Prevalences of adverse mental health symptoms

Adverse mental health symptoms	Overall		US		NY		LA		AU	
	(*N* = 5573)		(*N* = 3010)		(*N* = 507)		(*N* = 525)		(*N* = 1531)	
Anxiety Symptoms^a^										
Mean GAD-2 Score (SD)	1.59	1.810	1.60	1.847	1.64	1.799	1.61	1.745	1.57	1.759
No. with positive screens (%)	1303	(23.4)	712	(23.7)	129	(25.4)	124	(23.6)	338	(22.1)
Depression Symptoms^b^										
Mean PHQ-2 Score (SD)	1.39	1.750	1.33	1.749	1.43	1.651	1.49	1.780	1.49	1.780
No. with positive screens (%)	1172	(21.0)	617	(20.5)	115	(22.7)	105	(20.0)	335	(21.9)
Anxiety or Depression Symptoms										
No. with positive screens (%)	1622	(29.1)	872	(29.0)	162	(32.0)	150	(28.6)	438	(28.6)
Insomnia Symptoms^c^										
Mean SCI-02 Score (SD)	5.32	2.559	5.32	2.562	5.49	2.446	5.34	2.590	5.24	2.573
No. with positive screens (%)	1029	(18.5)	549	(18.2)	77	(15.2)	97	(18.5)	306	(20.0)

Survey responses are reported using descriptive statistics as indicated, including number of respondents (No.), percentage of respondents (%), mean, and standard deviation (sd)

^a^Symptoms of an anxiety disorder were assessed using the Generalized Anxiety Disorder 2-item (GAD-2) subscale of the Patient Health Questionnaire 4-item (PHQ-4). Respondents who scored ≥3 out of 6 on the GAD-2 were considered symptomatic

^b^Symptoms of a depressive disorder were assessed using the Patient Health Questionnaire 2-item (PHQ-2) subscale of the PHQ-4. Respondents who scored ≥3 out of 6 on the PHQ-2 were considered symptomatic

^c^Symptoms of insomnia were assessed using the Sleep Condition Indicator 2-item (SCI-02). Respondents who scored ≤2 out of 8 on the SCI-02 were considered symptomatic

Multivariable analysis of adverse mental health symptoms in the nation and cities models revealed that symptoms of anxiety or depressive disorders were more common among adults of female versus male gender (eg, cities model, aPR = 1.49, 95% CI = 1.23–1.81) and younger versus older age (eg, 18–24 versus ≥65 years, cities model, 3.28, 2.20–4.90), with all *p* ≤ 0.001 (Table 8). There were also differences by adherence with COVID-19 mitigation measures. In the nations model, symptoms of anxiety or depressive disorders were more common among those who reported being in quarantine or voluntarily spending the majority of time at home (1.77, 1.52–2.05 and 1.32, 1.14–1.53, respectively, both *p* < 0.001) versus those doing neither of these. The magnitudes of both aPRs were similar in the cities model, though adjusted prevalence of those spending the majority of time at home was not statistically significant. Very liberal respondents more commonly experienced anxiety or depressive disorder symptoms in both models. Insomnia symptoms were also more common among female versus male respondents (eg, cities model, 1.81, 1.35–2.42, *p* < 0.001), while the only difference by age group was observed among those aged 45–65 versus ≥65 years in the nations model (1.25, 1.04–1.49, *p* = 0.015). In the nations model but not the cities model, insomnia symptoms were more common among those who reported being in quarantine or voluntarily spending the majority of time at home (1.36, 1.13–1.65, *p* = 0.001 and 1.22, 1.02–1.46, *p* = 0.027, respectively) versus those doing neither of these. Statistically significant differences were not observed for adverse mental health symptoms regionally.

**Table 8:** Characteristics associated with adverse mental health symptoms

**For symptoms of an anxiety or depressive disorder**						
	**Nations (US and Australia [*n*** **= 4537])**			**Cities (New York and Los Angeles [*n*** **= 1032])**		
	**aPR**	**95% CI**	***P***	**aPR**	**95% CI**	***P***
Gender^a^ (reference: Male)						
Female	1.48	(1.34, 1.63)	< 0.001	1.49	(1.23, 1.81)	< 0.001
Age group, years (reference: ≥65)						
18–24	2.21	(1.85, 2.64)	< 0.001	3.28	(2.20, 4.90)	< 0.001
25–44	2.02	(1.72, 2.38)	< 0.001	2.78	(1.93, 3.99)	< 0.001
45–64	1.33	(1.12, 1.58)	0.001	2.07	(1.43, 2.98)	< 0.001
Political ideology (reference: Centre)						
Very Liberal	1.28	(1.12, 1.46)	< 0.001	1.38	(1.07, 1.80)	0.014
Slightly Liberal	1.00	(0.88, 1.14)	0.99	1.13	(0.86, 1.48)	0.38
Slightly Conservative	0.89	(0.77, 1.02)	0.099	1.15	(0.84, 1.58)	0.38
Very Conservative	0.94	(0.80, 1.10)	0.44	1.02	(0.71, 1.48)	0.90
Apolitical and/or prefer not to say	0.91	(0.77, 1.08)	0.28	1.12	(0.79, 1.58)	0.53
Region^b^ (reference: Australia and LA, respectively)						
US and NY, respectively	0.97	(0.88, 1.07)	0.49	1.13	(0.95, 1.35)	0.18
Self-reported quarantine or spending the majority of time at home (reference: No)						
Yes, Spending the majority of time at home	1.32	(1.14, 1.53)	< 0.001	1.22	(0.86, 1.74)	0.27
Yes, Quarantine	1.77	(1.52, 2.05)	< 0.001	1.52	(1.07, 2.15)	0.018
**For symptoms of insomnia**						
	**Nations (US and Australia [*n*** **= 4537])**			**Cities (New York and Los Angeles [*n*** **= 1032])**		
	**aPR**	**95% CI**	***P***	**aPR**	**95% CI**	***P***
Gender^a^ (reference: Male)						
Female	1.66	(1.46, 1.90)	< 0.001	1.81	(1.35, 2.42)	< 0.001
Age group, years (reference: ≥65)						
18–24	1.00	(0.79, 1.27)	0.98	0.73	(0.41, 1.31)	0.29
25–44	1.01	(0.84, 1.22)	0.92	1.02	(0.69, 1.52)	0.92
45–64	1.25	(1.04, 1.49)	0.015	1.09	(0.74, 1.59)	0.66
Political ideology (reference: Centre)						
Very Liberal	0.96	(0.78, 1.19)	0.71	1.16	(0.78, 1.73)	0.47
Slightly Liberal	0.95	(0.80, 1.13)	0.60	0.95	(0.64, 1.41)	0.79
Slightly Conservative	0.78	(0.65, 0.94)	0.011	1.02	(0.66, 1.59)	0.93
Very Conservative	0.98	(0.80, 1.20)	0.84	0.96	(0.58, 1.59)	0.87
Apolitical and/or prefer not to say	1.03	(0.83, 1.27)	0.82	0.86	(0.47, 1.59)	0.64
Region^b^ (reference: Australia and LA, respectively)						
US and NY, respectively	0.88	(0.77, 1.00)	0.058	0.83	(0.63, 1.08)	0.170
Self-reported quarantine or spending the majority of time at home (reference: No)						
Yes, Spending the majority of time at home	1.22	(1.02, 1.46)	0.027	1.04	(0.65, 1.67)	0.86
Yes, Quarantine	1.36	(1.13, 1.65)	0.001	1.31	(0.82, 2.10)	0.26

^a^For the multivariable analysis, respondents who reported a gender other than Male or Female (ie, “Other”, *n* = 4 [2 in the US sample, 2 in the Australian sample]) were excluded due to small cell sizes

^b^Regional reference groups were chosen to represent estimated prevalence ratios for dependent variables in high versus low SARS-CoV-2 prevalence regions

In addition to symptoms of anxiety, depression, and insomnia, many respondents reported COVID-19-specific concerns, as 4431 respondents (79.5%, range of samples, 77.5–82.1%) reported moderate to extreme concern about their own (61.9%) or others’ (75.5%) infection with SARS-CoV-2, and 3974 (71.3%, range of samples, 69.8–76.0%) reported similar concerns about their own (43.4%) or others’ (68.7%) death due to COVID-19 (Fig. 2). Access to testing (59.3%), medical care for COVID-19 (64.5%), medical care for pre-existing conditions due to hospital overload (59.2%), social or physical isolation (58.1%), and sense of purpose (49.8%) were also sources of moderate to extreme concern. Overall, 1217 respondents (21.8%) identified as high risk for severe COVID-19 infection. Across regions, nearly half (42.0–45.3%) reported spending considerable time (average, 23.2 h per week) consuming information (media, government reports, health officials, family) about COVID-19. Moreover, widespread concerns included the possibility of an economic recession and open-endedness of COVID-19 mitigation measures (79.2 and 72.2%, respectively) (Fig. 2).

Consistent across regions, respondents reported that COVID-19 and mitigation strategies have caused moderate to extreme disruption of social life (75.3%), family life (41.0%), work/studies (43.5%), productivity (41.6%), physical activity (45.1%), sexual activity (23.6%), and sleep patterns (39.3%) (Fig. 3). Overall, 1999 respondents (35.9%) reported exercising less frequently, and 409 (7.4%) reported concerning weight gain (Fig. 4). Daily outdoor light exposure was reduced by 1 h or more in 2279 respondents (40.9%). The estimated percentage of virtual interactions (versus face-to-face) increased from 14.6 to 66.1%, and 1786 respondents (32.0%) reported more than 1 h increase in daily screen time.

## Discussion

Resounding adherence with and support for strict COVID-19 mitigation measures was demonstrated in representative samples from the United States and Australia, despite the broad disruption these mitigation measures had on their social lives and daily routines, and their concerns about the economic consequences of such measures. Although 91.4% of respondents reported they believed they would never be infected with SARS-CoV-2 (range of samples, 89.2–92.6%), controlling COVID-19 was a top public priority at the outset of the pandemic. Contrary to negative public attitudes about and low adherence with recommended mitigation during the last pandemic [17, 18] declared by the World Health Organization for novel influenza A (H1N1) in 2009 [19], the initial public response to the COVID-19 pandemic represented a hitherto unprecedented level of adherence with public health emergency measures that has had and will continue to have a profound impact on economics and public life.

These results demonstrate an enhanced public adherence with stay-at-home orders in the US compared to reported adherence during the weeks before such orders were initially widely implemented [20]. Recently published data from a convenience sample suggest that one month later (May 2020), nearly half of adults in the UK were intentionally non-adherent with government-imposed mitigation measures [21]. Differences in the survey sampling methodology, the questions used to assess adherence with mitigation policies, recruitment timeframe, and study populations make it difficult to make direct comparisons of these results, however, which are not consistent with our findings in May 2020 among US adults, who reported sustained adherence to and support for stay-at-home orders and nonessential business closures [22]. Our findings represent one of the earliest assessments of mental health and life impact of the COVID-19 pandemic and its mitigation, having been administered in early April 2020, near the onset of initial stay-at-home orders in the US and Australia. They reveal that the adverse life impact and mental health symptoms observed throughout the pandemic—including significant disruption of daily life and two- to three-fold increased prevalences of anxiety and depressive disorder symptoms compared with pre-pandemic estimates [23–31]—were evident within a month after the pandemic was declared by the WHO, in regions and countries with both high and low prevalences of COVID-19. These broad impacts of the COVID-19 pandemic and its mitigation are similar to those observed during previous infectious disease outbreaks [32–34]. These findings may also provide insight into behavioural countermeasures related to sleep, exercise, and diet that may reduce adverse health consequences of COVID-19 mitigation measures.

Strengths of this study include rapid and largescale assessment of public adherence, priorities, and life impacts related to COVID-19 and its mitigation in representative samples from developed nations and cities with high and low SARS-CoV-2 prevalences near the onset of the pandemic and widespread stay-at-home orders, enabling comparisons across jurisdictions at a simultaneous timepoint using consistent questions. Limitations include self-report measures of behaviours, which are subject to recall, response, and social desirability biases. Survey samples also have potential non-response and self-selection biases among respondents, and while quota sampling was used to improve sample representativeness in each region, Internet-based samples may not fully represent the 2020 US and Australian populations. However, the high response rate (63.9%) and consistency of responses across cities and countries despite vastly different rates of SARS-CoV-2 infection, governments, and mitigation strategies support the robustness of our findings.

As controversies over the legality [35] and balance between duration and nature of mitigation strategies and related consequences mounted following their implementation in the second quarter of 2020, with the prospect of repeated and protracted stay-at-home orders being recommended over the next 2 years [36], rigorous assessment of public priorities, adherence, and life impact will be paramount. Over the past year, Australia capitalized on the broad support for stringent mitigation measures documented herein, implementing widescale testing, contact tracing, and, in some cases, strict mitigation measures (eg, mandatory mask usage in public, physical distancing, and quarantining as necessary to contain regional outbreaks). In contrast, the United States did not capitalize on this broad initial support for stringent mitigation measures, which were effective in reducing community mobility [37] and slowing community transmission of SARS-CoV-2 [38]. Jurisdictions across the US opted instead to lift restrictions, which was associated with increased mobility [39], before testing for SARS-CoV-2 infection was readily available and widespread community transmission of COVID-19 was contained. These are among policies that a recent *Lancet Commission* deemed to have substantially contributed to excess preventable COVID-19 deaths in the US compared with other high-income countries [40]. Notably, as of December 27, 2020, the cumulative COVID-19 death rate in Australia was 3.6 deaths per 100,000 population, with 0 new deaths in the prior week, and the COVID-19 death rate in the United States was 99.1 deaths per 100,000 population, with 16,864 new deaths in the prior week (5.1 deaths per 100,000 population) [41]. The weekly death rate in the US in the last full week of December was more than 40% greater than the cumulative per capita death rate during the entire pandemic in Australia.

## Conclusions

In early April 2020, within 1 month of the declaration of the COVID-19 pandemic, US and Australian adults reported widespread adherence with stringent mitigation policies, and strongly supported continued government-imposed stay-at-home orders for as long as necessary to contain the COVID-19 pandemic, despite the considerable sacrifices that these measures required, and the potentially significant economic consequences. Markedly elevated prevalences of adverse mental health symptoms compared to pre-pandemic estimates were found in both nations and cities, and an extensive degree of life disruption attributed to COVID-19 was documented. These data highlight that respondents of younger age, female gender, and those in quarantine or spending most of the time at home more commonly experienced anxiety and depression symptoms than persons of other demographic groups, regardless of whether they were in regions with high or low SARS-CoV-2 prevalence. Timely dissemination of routine surveillance of public attitudes, behaviours, and beliefs regarding mitigation measures that require public support and adherence is important to inform strategies to improve adherence. They further underscore the importance of assessment of the potential life and mental health impacts of the pandemic and its mitigation, and may be used to inform policymakers during both the current and future infectious disease outbreaks.

## Supplementary Information

**Additional file 1.** Respondent 2019 Place of Residency in Nationwide Samples. Description of data: Respondents reported their primary place of residence between October and December 2019. For the nationwide US sample, the distribution of respondents among the fifty states and Washington District of Columbia are reported in comparison to population estimates from the US Census Bureau as of July 2019 [42]. For the nationwide AU sample, the distribution of respondents among the six states and two internal territories are reported in comparison to population estimates from the AU Bureau of Statistics as of September 2019 [43]. In total, 44/4541 respondents (0.97%) lived outside of the US or AU between October and December 2019 and were currently residing in these regions. These data support the nationwide samples as geographically representative by state or territory.

## Data Availability

The datasets used and/or analysed during the current study are available from the corresponding author upon reasonable request.

## Abbreviations

AU: Australia
COVID-19: Coronavirus disease 2019
GAD-2: 2-item Generalized Anxiety Disorder
HITS: Hurt-Insult-Threaten-Scream
H1N1: Novel influenza A
LA: Los Angeles metropolitan area
MEQ: Morningness-Eveningness Questionnaire
No.: Number
NY: New York City metropolitan area
OSA50: Obstructive Sleep Apnoea 50
PHQ-2: 2-item Patient Health Questionnaire
PHQ-4: 4-item Patient Health Questionnaire
PSS-4: 4-item Perceived Stress Scale
SCI-02: Short-Form Sleep Condition Indicator
sd: Standard deviation
μMCTQ: μshort Munich ChronoType Questionnaire
US: United States of America
