# Supplementary material for: Early public adherence with and support for stay-at-home COVID-19 mitigation strategies despite adverse life impact: a transnational cross-sectional survey study in the United States and Australia
Source: BMC Public Health. 2021 Mar 15;21:503. doi: 10.1186/s12889-021-10410-x (PMC7957462; doi:10.1186/s12889-021-10410-x)
Supplement: Supplementary file 1 — Additional file 1. Respondent 2019 Place of Residency in Nationwide Samples. Description of data: Respondents reported their primary place of residence between October and December 2019. For the nationwide US sample, the distribution of respondents among the fifty states and Washington District of Columbia are reported in comparison to population estimates from the US Census Bureau as of July 2019 [42]. For the nationwide AU sample, the distribution of respondents among the six states and two internal territories are reported in comparison to population estimates from the AU Bureau of Statistics as of September 2019 [43]. In total, 44/4541 respondents (0.97%) lived outside of the US or AU between October and December 2019 and were currently residing in these regions. These data support the nationwide samples as geographically representative by state or territory. [file 12889_2021_10410_MOESM1_ESM.pdf]

US Census Bureau Nationwide Population Place of Residence: July 2019			US Sample Respondent Place of Residence: September to December 2019			Comparison
**State or Territory**	**Population**	**% of US Population**	**State or Territory**	**Respondents**	**% of US Sample**	**Difference in % of US Population**
Alabama	4903185	1.49%	Alabama	41	1.36%	-0.13%
Alaska	731545	0.22%	Alaska	9	0.30%	0.08%
Arizona	7278717	2.22%	Arizona	100	3.32%	1.10%
Arkansas	3017804	0.92%	Arkansas	10	0.33%	-0.59%
California	39512223	12.04%	California	241	8.01%	-4.03%
Colorado	5758736	1.75%	Colorado	65	2.16%	0.41%
Connecticut	3565287	1.09%	Connecticut	43	1.43%	0.34%
Delaware	973764	0.30%	Delaware	15	0.50%	0.20%
District of Columbia	705749	0.22%	District of Columbia	6	0.20%	-0.02%
Florida	21477737	6.54%	Florida	294	9.77%	3.22%
Georgia	10617423	3.23%	Georgia	105	3.49%	0.25%
Hawaii	1415872	0.43%	Hawaii	27	0.90%	0.47%
Idaho	1787065	0.54%	Idaho	19	0.63%	0.09%
Illinois	12671821	3.86%	Illinois	115	3.82%	-0.04%
Indiana	6732219	2.05%	Indiana	49	1.63%	-0.42%
Iowa	3155070	0.96%	Iowa	36	1.20%	0.23%
Kansas	2913314	0.89%	Kansas	22	0.73%	-0.16%
Kentucky	4467673	1.36%	Kentucky	40	1.33%	-0.03%
Louisiana	4648794	1.42%	Louisiana	28	0.93%	-0.49%
Maine	1344212	0.41%	Maine	17	0.56%	0.16%
Maryland	6045680	1.84%	Maryland	46	1.53%	-0.31%
Massachusetts	6892503	2.10%	Massachusetts	85	2.82%	0.72%
Michigan	9986857	3.04%	Michigan	96	3.19%	0.15%
Minnesota	5639632	1.72%	Minnesota	50	1.66%	-0.06%
Mississippi	2976149	0.91%	Mississippi	17	0.56%	-0.34%
Missouri	6137428	1.87%	Missouri	51	1.69%	-0.18%
Montana	1068778	0.33%	Montana	7	0.23%	-0.09%
Nebraska	1934408	0.59%	Nebraska	20	0.66%	0.08%
Nevada	3080156	0.94%	Nevada	57	1.89%	0.96%
New Hampshire	1359711	0.41%	New Hampshire	16	0.53%	0.12%
New Jersey	8882190	2.71%	New Jersey	27	0.90%	-1.81%
New Mexico	2096829	0.64%	New Mexico	19	0.63%	-0.01%
New York	19453561	5.93%	New York	110	3.65%	-2.27%
North Carolina	10488084	3.20%	North Carolina	92	3.06%	-0.14%
North Dakota	762062	0.23%	North Dakota	5	0.17%	-0.07%
Ohio	11689100	3.56%	Ohio	114	3.79%	0.23%
Oklahoma	3956971	1.21%	Oklahoma	28	0.93%	-0.28%
Oregon	4217737	1.28%	Oregon	59	1.96%	0.68%
Pennsylvania	12801989	3.90%	Pennsylvania	190	6.31%	2.41%
Rhode Island	1059361	0.32%	Rhode Island	13	0.43%	0.11%
South Carolina	5148714	1.57%	South Carolina	51	1.69%	0.13%
South Dakota	884659	0.27%	South Dakota	7	0.23%	-0.04%

State or Territory	Population	% of US Population	State or Territory	Respondents	% of US Sample	Difference in % of US Population
Tennessee	6829174	2.08%	Tennessee	58	1.93%	-0.15%
Texas	28995881	8.83%	Texas	198	6.58%	-2.26%
Utah	3205958	0.98%	Utah	29	0.96%	-0.01%
Vermont	623989	0.19%	Vermont	6	0.20%	0.01%
Virginia	8535519	2.60%	Virginia	75	2.49%	-0.11%
Washington	7614893	2.32%	Washington	90	2.99%	0.67%
West Virginia	1792147	0.55%	West Virginia	14	0.47%	-0.08%
Wisconsin	5822434	1.77%	Wisconsin	75	2.49%	0.72%
Wyoming	578759	0.18%	Wyoming	3	0.10%	-0.08%
			Outside US in Sept to Dec 2019 (currently in US)	20	0.66%	
**Total US population**	**328239523**		**Total US Sample Size**	**3010**		

AU Bureau of Statistics Nationwide Population Place of Residence: September 2019			AU Sample Respondent Place of Residence: September to December 2019			Comparison
**State or Territory**	**Population**	**% of AU Population**	**State or Territory**	**Population**	**% of AU Sample**	**Difference in % of AU Population**
New South Wales	8118000	31.89%	New South Wales	379	24.76%	-7.13%
Queensland	5115500	20.09%	Queensland	278	18.16%	-1.93%
South Australia	1756500	6.90%	South Australia	201	13.13%	6.23%
Tasmania	535500	2.10%	Tasmania	58	3.79%	1.69%
Victoria	6629900	26.04%	Victoria	335	21.88%	-4.16%
Western Australia	2630600	10.33%	Western Australia	212	13.85%	3.51%
Northern Territory	245600	0.96%	Northern Territory	33	2.16%	1.19%
Australian Capital Territory	428100	1.68%	Australian Capital Territory	11	0.72%	-0.96%
			Outside AU Sept to Dec 2019 (currently in AU)	24	1.57%	
**Total AU population**	**25459700**		**Total AU Sample Size**	**1531**		
